# Discrimination and Bias in State Triage Protocols Toward Populations With Intellectual Disabilities During the COVID-19 Pandemic

**DOI:** 10.1017/dmp.2021.81

**Published:** 2021-03-25

**Authors:** Ashley Brooke Felt, Dionne Mitcham, Morgan Hathcock, Raymond Swienton, Curtis Harris

**Affiliations:** 1 Institute for Disaster Management, College of Public Health, University of Georgia, Athens, GA, USA; 2 Department of Emergency Medicine, University of Texas Southwestern Medical Center, Dallas, TX, USA

**Keywords:** COVID-19, disabilities, discrimination, social work, triage

## Abstract

Individuals with intellectual disabilities face discrimination on a daily basis. The coronavirus disease (COVID-19) pandemic has highlighted the systemic ableism that is embedded within American culture, particularly through health care bias and discrimination. In turn, this creates further marginalization during diagnosis, triage, and treatment of the novel coronavirus. Multiple states have filed complaints against state triage protocols that suggest an abled life is more worthy than a life with a disability. Although many of these protocols have been updated and replaced, generalized triage statements fail to address health care bias that is embedded within the American system. In addition to the existing solutions, proposed solutions to addressing health care bias include integrating social workers into the emergency management process and the overall disaster management field. To combat bias and ableism across the health care system, a social justice perspective that highlights discrimination, inequalities, and inequities in overall individual care must be adopted.

## Introduction and Problem Statement

State emergency triage protocols are necessary to have in place for circumstances where the present needs exceed the available resources. While these protocols are crucial for health care systems, there have been policies established in various states that discriminate against people living with intellectual disabilities (PWID), inherently adding to bias in the medical treatment being provided and the overall allocation of scarce resources. Unfortunately, having a disability is not only a matter of the body in the United States, but also ours is a culture of systemic ableism that has ebbed and flowed throughout history and is now re-emerging during the 2019 coronavirus disease (COVID-19) pandemic. Multiple disability advocacy groups have filed complaints to the US Department of Health and Human Services against their state emergency triage protocols, which allow clinicians to not only discern the allocation of lifesaving resources – such as ventilators – on the basis of disability, but also, in some cases, withdraw necessary care from individuals to whom those resources are already allocated.^1,2^ The emergence of this problem now, in the face of the COVID-19 pandemic, is indicative of the warped perception of PWID within the health care system.

## Evidence

Advancements in modern technology have extended the life expectancies for PWID, enabling even the most severely affected to live happy, independent lives.^3^ However, research has demonstrated that people with disabilities have a disproportionate exposure to disasters,^4,5^ with COVID-19 being no exception. According to the American Association on Intellectual and Developmental Disabilities (AAIDD), an intellectual disability (ID) is that which poses limitations to an individual’s “intellectual functioning and adaptive behavior” capabilities.^6^ The prevalence of IDs worldwide is estimated to be about 1%.^7^ Currently, there are over 25 million cases of COVID-19 in the United States.^8^ COVID-19 has disproportionately affected the elderly and individuals with underlying comorbidities. According to the Centers for Disease Control and Prevention (CDC), adults with disabilities are 3 times more likely to have “heart disease, stroke, diabetes, or cancer” compared with adults without disabilities in the United States.^9^ PWID with these comorbidities may have a higher likelihood of contracting COVID-19 and suffering from more severe symptoms; however, those without comorbidities are not automatically more likely to catch the virus or die from it. It is imperative that patients be evaluated objectively on a case-by-case basis, rather than inherently classified as less likely to recover or have sufficient quality of life based on their disability status. According to the Alabama Disabilities Advocacy Program, failing to do so violates several State and Federal statutes:

In accordance with the Americans with Disabilities Act, Section 504 of the Rehabilitation Act, and Section 1557 of the Affordable Care Act, decisions about how treatment should be allocated must be made based on individualized determinations, using current objective medical evidence, and not based on generalized assumptions about a person’s disability.^2(p2)^

### State Triage Protocols

Presently, complaints against triage protocols have been filed in multiple states, including Alabama, Kansas, and Washington, among others.^2^ For brevity, we will examine the case of Alabama. The complaint filed by the Alabama Disabilities Advocacy Program (ADAP) in March, 2020, quotes a document titled, “Criteria for Mechanical Ventilator Triage Following Proclamation of Mass-Casualty Respiratory Emergency,” issued in 2010 under the Alabama Department of Public Health (ADPH). The complaint states that the document violates sections of the Rehabilitation Act, the Affordable Care Act (ACA), and the American Disabilities Act (ADA).^2^ The protocol outlines a tiered approach to rationing ventilator access when resources become scarce, basing exclusion criteria on the presentation of end-stage organ failure. Appendix 2 of the plan clarifies the conditions that constitute end-stage organ failure by organ system; under the subsection, “Neurological,” it reads:

*For example, persons with severe mental retardation, advanced dementia or severe traumatic brain injury may be poor candidates for ventilator support. The average life expectancy of persons with mental retardation now spans to the seventh decade and persons with significant neurological impairments can enjoy productive happy lives. Functional assessment for persons with intellectual disability, complex neurological problems, dementia, or mixtures of symptoms should focus on premorbid function in all domains of life including social, intellectual, professional, etc.* ^2*(p*8*)*^

First, the statement that PWID have comparable life expectancies and can lead fulfilling lives directly opposes the following statement that clinicians should consider their social and professional lives when determining whether they are eligible for lifesaving care. Furthermore, in an emergency scenario where family members or caretakers may not be allowed to accompany patients to an exam room, or where alternative communicative devices may be unavailable, non-verbal individuals may be completely unable to communicate their needs and medical history to their physician. In this case, the quality of life of the patient is subjective to the clinician who could struggle to fully understand the patient’s own views on her or his own quality of life. Further complications arise when we consider that, when health care systems are overwhelmed, medical records may not be immediately available during triage and intake exams, so the knowledge of pre-morbidities for all patients is limited. Ultimately, it boils down to the harsh reality: If a decision is to be made between 2 patients with similar presentations of symptoms, some triage protocols direct that an abled life is more worthy than a life with a disability.

The ADPH Center for Emergency Preparedness web page claims that the referenced document is no longer in effect and has been replaced by the Alabama Crisis Standards of Care Guidelines, dated February 28, 2020.^10^ The new document states the importance of equity and non-discrimination numerous times; however, it provides no guidelines or specific alterations to the mass casualty protocol that it is intended to replace.^11^ Under the subsection, “Stewardship of Resources,” the following statement is provided:

*When an extreme disaster overwhelms healthcare resources, priority should be given to patients whose lives would most likely be saved and patients whose outcomes would most likely improve. Those patients should be given priority over patients who would likely die even with treatment and patients who would likely survive without treatment.* ^11*(p*14*)*^

While conceptually sound, generalized statements such as these do not address the biases against those PWID within the health care system, wherein individuals with disabilities are presumed to be less likely to survive.

## Alternative Policy Responses

### Existing Solutions

People with disabilities have been mistreated and discriminated against throughout history.^3^ To promote social justice and prevent further mistreatment of an entire population, protections for PWID need to be integrated into the COVID-19 response. The Department of Health and Human Services has provided guidance for health professionals on best practices to avoid disability-based treatment rationing.^12^ Non-discrimination clauses and recognition of treatment bias are recommendations for state hospitals to include in their allocation of treatment and triage plans.^12^ Bias in treatment needs to be addressed to prevent discrimination in the triage process.

Despite the advancements in adaptive devices and assistive technology, the inclusion of such essential tools supporting communication and activities of daily living is lacking in emergency management planning.^13^ The importance of supporting all persons with any access or functional assistance needs from any form of impairment or disability (eg, motor, sensory, cognitive, behavioral) with appropriate devices and technology must be a priority in public health preparedness and planning. Additionally, health care staff need training on the rights and needs of PWID to ensure unbiased and adequate treatment.^14^ Past recommendations for addressing health care inequalities prior to COVID-19 include training health care professionals on the diverse needs of PWID; training individuals with disabilities, their families, and community support staff on best communication practices and advocacy for health care needs; improving access to quality health care at health practices and hospitals; and reducing insurance reimbursement barriers for health care providers for additional time and resources.^15^

Human rights-based solutions have been proposed to address the rights of children with disabilities during the COVID-19 pandemic, but this kind of approach should be applied to adults with IDs as well. Successful, inclusive community-based responses need to include PWID in the planning process to account for the unique needs of this population.^16^ To have a plan that represents all community members, a diverse group of people should be included (ie, the Whole Community Concept).^17^ All populations and stakeholders in the community need to have a seat at the table. A community-based initiative focused to include PWID in relevant preparedness planning and decision-making leads to improved engagement and overall disaster preparedness for this population.^18^ Policy change that addresses everyone with a disability or access and functional need is important to building community resilience.^19^

### Proposed Solutions

In addition to the existing solutions, it is necessary to integrate social workers into the emergency response process. As a profession, social workers value service, social justice, dignity and worth of the person, importance of human relationships, integrity, and competence.^20^ Addressing discrimination, inequality, and inequities is at the core of social work practice, while also valuing and respecting the individual’s right to self-determination. PWID have a right to receive unbiased health care treatment, especially at a time when this population feels largely marginalized and overlooked. The integrated response process includes using medical social workers in hospital settings as advocates for incoming patients seeking care for COVID-19 and ensuring that PWID receive non-discriminatory treatment. Bias in health care is often unconscious and results in increasing systemic barriers. Assigning social workers as advocates will promote social justice and alleviate the burden of inequality felt by this population.

As a long-term solution, social workers need to be more integrated into the disaster management field. Including social workers in disaster protocol and planning will increase the inclusion of PWID and ensure that their needs are met and accounted for in all disaster scenarios. In health-related disasters, such as COVID-19, social disparities are highlighted and disproportionately impact certain populations over others. This is more than a response issue; it is a social justice issue that needs to be addressed at the root cause. To mitigate social disparities and alleviate the disproportionate burden on marginalized populations, social workers and emergency managers must collaborate together. Bias toward PWID must be addressed in health care and across professions.

## Recommendation and Conclusion

Based on this policy analysis, the recommended alternative for better treatment of PWID in health-related disasters is to integrate the social workers into the emergency management field. Historically, PWID have had difficulty accessing daily health care services compared with individuals without disabilities, which is further heightened due to the COVID-19 pandemic.^21^ PWID face challenges when accessing health care due to a lack of social support and biases of health care workers.^21^ With our suggested solutions, we can work toward integrating the field of social work with disaster protocol, planning, and management.

*Each and every person in the United States has certain rights, not just those who make a threshold showing sufficient intellectual ability. When medical professionals and others fail to accept the rule of law, there is a dissonance between clinical practice and individual rights.* ^3^
